# Photo-induced non-volatile VO_2_ phase transition for neuromorphic ultraviolet sensors

**DOI:** 10.1038/s41467-022-29456-5

**Published:** 2022-04-01

**Authors:** Ge Li, Donggang Xie, Hai Zhong, Ziye Zhang, Xingke Fu, Qingli Zhou, Qiang Li, Hao Ni, Jiaou Wang, Er-jia Guo, Meng He, Can Wang, Guozhen Yang, Kuijuan Jin, Chen Ge

**Affiliations:** 1 Beijing National Laboratory for Condensed Matter Physics, Institute of Physics, Chinese Academy of Sciences, 100190 Beijing, China; 2 University of Chinese Academy of Sciences, 100049 Beijing, China; 3 College of Science, China University of Petroleum (East China), 266580 Qingdao, China; 4 Key Laboratory of Terahertz Optoelectronics, Ministry of Education, and Beijing Advanced Innovation Center for Imaging Theory and Technology, Department of Physics, Capital Normal University, 100048 Beijing, China; 5 College of Physics, University-Industry Joint Center for Ocean Observation and Broadband Communication, State Key Laboratory of Bio-Fibers and Eco-Textiles Qingdao University, 266071 Qingdao, China; 6 Beijing Synchrotron Radiation Facility, Institute of High Energy Physics, Chinese Academy of Sciences, 100049 Beijing, China

**Keywords:** Electronic properties and materials, Sensors and biosensors

## Abstract

In the quest for emerging in-sensor computing, materials that respond to optical stimuli in conjunction with non-volatile phase transition are highly desired for realizing bioinspired neuromorphic vision components. Here, we report a non-volatile multi-level control of VO_2_ films by oxygen stoichiometry engineering under ultraviolet irradiation. Based on the reversible regulation of VO_2_ films using ultraviolet irradiation and electrolyte gating, we demonstrate a proof-of-principle neuromorphic ultraviolet sensor with integrated sensing, memory, and processing functions at room temperature, and also prove its silicon compatible potential through the wafer-scale integration of a neuromorphic sensor array. The device displays linear weight update with optical writing because its metallic phase proportion increases almost linearly with the light dosage. Moreover, the artificial neural network consisting of this neuromorphic sensor can extract ultraviolet information from the surrounding environment, and significantly improve the recognition accuracy from 24% to 93%. This work provides a path to design neuromorphic sensors and will facilitate the potential applications in artificial vision systems.

## Abstract (web summary)

Bioinspired neuromorphic vision components are highly desired for the emerging in-sensor computing technology. Here, Ge et al. develop an array of optoelectronic synapses capable of memorizing and processing ultraviolet images facilitated by photo-induced non-volatile phase transition in VO_2_ films.

## Introduction

Visual input, as one of our most important sensory functions, plays a critical role in human perception. More than 80% of the information received from the external environment is from vision^1–3^. Human vision is fundamentally a memory-based process, as the sensory neurons in the retina can not only detect light signals, but they also preform image preprocessing before more complicated visual information processing takes place in the visual cortex^4,5^. Existing CMOS-based artificial intelligence vision systems are composed of a photoreceptive chip, an analog-to-digital converter that transforms electrical input into digital signals, and an external artificial neural network (ANN) that preforms complex image processing tasks^4,6^. However, the physical separation of the functional components generates a large amount of redundant data during storage and transfer processing, which in turn leads to delays in data access and high power consumption. In addition, with the rapid growth of sensory nodes, bandwidth limitations make it difficult to send all data back to central or cloud computers quickly to realize real-time processing^1,4,7,8^. For this reason, the development of multifunctional electronic devices integrating sensing, memory, and processing functions is an effective way to improve the efficiency of artificial vision systems^9^. Optoelectronic neuromorphic sensors with both the sensing characteristics for light stimulation and nonvolatile multi-level storage characteristics provide a good choice for the development of artificial vision systems. Recent studies have shown that these sensors can perform image preprocessing and neuromorphic computing functions for machine vision systems^4,6,10^.

Most of the reported studies focus on the development of neuromorphic sensors operating in visible range, which are by design aimed to be alternatives to the human visual system. To ensure their survival and reproduction, most animal species have the capability of recognizing and perceiving ultraviolet (UV) light. For example, bees have developed an amazing ability to navigate and locate flowers using their UV-sensitive visual and nervous systems^3^, while reindeer can identify ground moss under snow in faint light by perceiving the intensity of the reflected UV light^11^. On the other hand, depending on its intensity, duration, and frequency of exposure and other factors, UV light can cause premature aging, skin cancer, macular degeneration, cataracts, and other ailments^12,13^. Since human beings cannot perceive this wavelength, the development of UV neuromorphic sensors can complement humans’ understanding of UV light and be instrumental for different applications such as biological sensors, healthcare devices, rocket early warning and missile detection^11,14^. However, the reported UV optoelectronic synapses are mainly based on the charge trapping/detrapping effect, which results in large writing non-linearity. Moreover, since these devices need to separate the photo-generated electron-hole pairs to achieve non-volatile memory characteristics, they are generally arranged as multi-layer structures, which increases the difficulty of large-scale industrial fabrication. Materials that respond to UV stimuli in conjunction with non-volatile phase transformation could open new avenues for the realization of high-performance neuromorphic sensors.

As an archetypal Mott material, vanadium dioxide (VO_2_) undergoes a typical phase transition from the low-temperature monoclinic (M_1_) phase to the high-temperature rutile (R) phase at the critical temperature of ~341 K^15,16^. During the phase transition process, VO_2_ exhibits a sharp change of resistance with several orders of magnitude and a pronounced optical switching in the infrared region. Benefitting from this phase transition, VO_2_ has been widely exploited in novel electronic and optical applications such as smart windows^17–19^, bolometers for infrared detection^20–22^, switching devices^23–25^, and neuromorphic devices^26–28^. In particular, optical control of VO_2_’s phase transition at room temperature has great potential for investigating the intrinsic physical mechanism and realizing optical modulation devices. Currently, optical pumping is used to induce the photoexcitation insulator-metal phase transition, which promises to allow vital insights into the nature of each state and may lead to metastable new phases under non-equilibrium conditions^29–32^. However, being an ultrafast excitation, such optical means cannot introduce stable phase transition; instead, a transient process on the picosecond scale is induced. Several works show that the electrical properties of VO_2_ can be modulated through various means of irradiation, such as electron beams^33^, X-ray^34^, and even UV light^35^. These results indicate the possibility of photo-controlled phase transition in VO_2_.

In this study, we present a novel neuromorphic sensor based on the optical control of phase transition in VO_2_ films with UV light, and demonstrate that this device can realize UV light perception and multi-level storage functions. The proportion of monoclinic phase in the film decreases with UV radiation dose, indicating the tunability of the phase transformation introduced by the optical stimulation. Based on this mechanism, the optoelectronic synaptic functions with integrated sensing and non-volatile multilevel storage features are successfully realized in VO_2_ grown on both Al_2_O_3_ and Si substrates. Using the optoelectronic synapses as sensing units, an ANN is constructed to realize the image sensing and memorization functions. The neuromorphic sensor array can extract the UV information from the surrounding environment, which significantly improves the image recognition rate on the MNIST handwritten dataset from 24% to 93%.

## Results

### Light-dosage-dependent synaptic plasticity

**Fig. 1:**
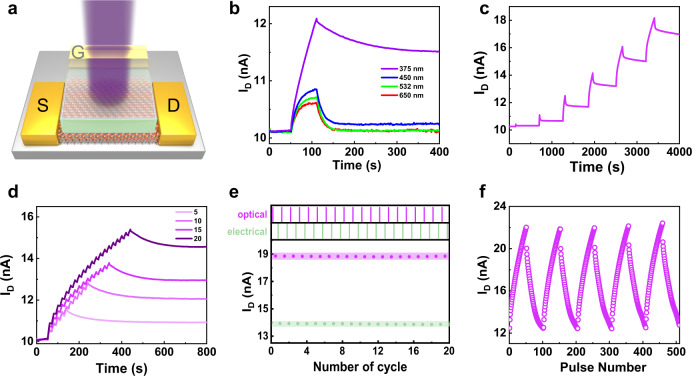
Optical sensing and non-volatile multi-level storage characteristics. **a** Schematic illustration of the neuromorphic transistor stimulated using 375 nm UV light. The VO_2_ film serves as a channel between the source (S) and drain (D) electrodes, and ionic liquid is used as a gating medium. **b** *I*_D_ as a function of time under different light wavelengths (64 mW/cm^2^ for 60 s). **c** *I*_D_ response to UV irradiation at different durations (84 mW/cm^2^ for 1 s, 10 s, 50 s, 100 s, 150 s, 200 s). **d** Spike-number-dependent plasticity under UV irradiation (84 mW/cm^2^ for 10 s). **e** Pulse-switching characteristics. The optical potentiation (84 mW/cm^2^ for 20 s) and electrical depression (−2.5 V for 20 s) were used for the switching process. *I*_D_ was read 2 s after the light and voltage pulse stimuli. **f** Light-controlled LTP (light intensity 84 mW/cm^2^ for 10 s, spaced 10 s apart) and *V*_G_-controlled LTD (−1.5 V to −3.5 V, duration of 10 s, spaced 10 s apart) for 50 pulses. A constant source-drain voltage *V*_SD_ = 50 mV was applied to monitor the channel current.

We grew epitaxial VO_2_ films with a transition temperature of about 341 K on r-Al_2_O_3_ substrates using pulsed laser deposition (PLD) technique. The high quality of VO_2_ films was confirmed using an atomic force microscope image (AFM) and through its X-ray diffraction (XRD) pattern (Supplementary Figure 1). Then, we fabricated the film into an optoelectronic transistor. The schematic diagram of the device structure is shown in Fig. 1a. More details about the device fabrication can be found in the Methods Section. Ohmic contact was exhibited between the source and drain electrodes (Supplementary Fig. 2). The temporal changes in the drain currents *I*_D_ were measured under red (650 nm), green (532 nm), blue (450 nm), and UV (375 nm) light at an intensity of 64 mW/cm^2^. As shown in Fig. 1b, the transistor exposed to UV light exhibits non-volatility, while the *I*_D_ irradiated under visible light returned to its initial state. The different behaviors of *I*_D_ under visible and UV light are due to the different modulation mechanisms, which will be discussed in detail further below. Moreover, we investigated the effect of the light exposure on the channel current at different wavelength (Supplementary Fig. 3). As the illumination intensity increased, so did the photocurrent; however, only the device illuminated using UV light exhibited obvious non-volatile behavior. The transistor also exhibited weak non-volatile tunability under blue light, which can be due to the larger photon energy compared to the other visible lights used. It should be noted that this change was very small compared with that of UV illumination. In order to verify that the non-volatility is only dependent on the light wavelength, we irradiate the device under a stronger light intensity with 550 mW/cm^2^ at 532 nm (Supplementary Fig. 4). Although the device takes a longer relaxation time, it will eventually return to the initial state, showing a volatile characteristic. Since the transistor exhibited a synaptic property under UV exposure, we emulated other basic features of synaptic plasticity to simulate the learning and memory functions.

Figure 1c shows the stepwise increase of *I*_D_ under illumination for six different durations using a constant light intensity of 84 mW/cm^2^. The durations were 1 s, 10 s, 50 s, 100 s, 150 s, 200 s, respectively, and the channel current was monitored at a small *V*_D_ of 50 mV. The result indicated that *I*_D_ increased along with the increase of exposure duration and good stability was demonstrated in each state. Then, we chose 10 s as the light pulse width while keeping the other conditions fixed, and measured the excitatory postsynaptic current (EPSC) response of neuromorphic transistor at different pulse numbers and different pulse intervals (Fig. 1d and Supplementary Fig. 5). It is found that both a pulse number increase and a pulse interval decrease lead to a significant enhancement of the synaptic strength. Furthermore, the pulse-switching characteristics of optical potentiation (light intensity of 84 mW/cm^2^, duration of 20 s) and electrical depression (voltage of −2.5 V, duration of 20 s) was studied in Fig. 1e. The channel current of the transistor can be reversibly switched between high- and low-current states dozens of times without significant degradation. The long-term synaptic plasticity, which includes the long-term potentiation (LTP) and long-term depression (LTD), was also simulated using our transistor (Fig. 1f). We applied 50 consecutive photonic pulses at an intensity of 84 mW/cm^2^ and a pulse duration of 10 s to emulate LTP. In contrast, the LTD appeared when 50 V_G_ pulses were applied to the gate electrode (voltage varying from −1.5 V to −3.5 V, duration of 10 s). Here, electrolyte gating was utilized to achieve low voltage regulation due to its electric double layer effect, which can reduce device energy consumption effectively^27,36^. The results show that under optical writing and electrical erasing for programming, the device can be controlled continuously and in an adjustable multi-state non-volatile manner. The non-linearity values of potentiation and depression were calculated as 0.2 and 1.1, respectively. More details about the calculation formulas of non-linearity values and corresponding fitting parameters can be found in Supplementary Note 1 and Supplementary Table 1. Obviously, LTP exhibited high linearly, while LTD exhibited a decrease in linearity due to factors such as the internal dynamics of the ionic liquid. The non-linearity factors of this VO_2_-based neuromorphic transistor were significantly lower compared with those reported in previous works (Supplementary Table 2). In order to ensure good stability at each state, we examined the retention characteristics after writing and erasing operations (Supplementary Fig. 7), where it was found that the channel current remained constant for at least 4000 s after each operation.

**Fig. 2:**
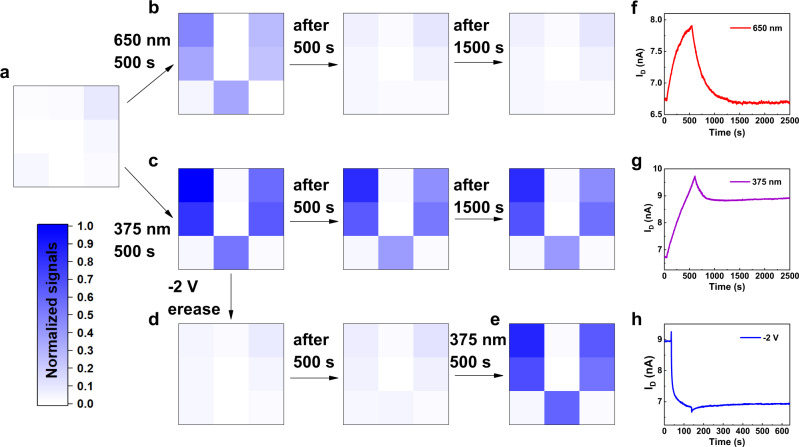
Selective recognition of UV light and image memorization. **a** Schematic structure of a 3 × 3 neuromorphic transistor array. **b**, **c** illustrate the image memory of the letter V under 650 nm and 375 nm, respectively, at 64 mW/cm^2^ for 500 s. Retention characteristics were also investigated at 1000 s and 2500 s. **d** Electrical erasing process (−2 V for 100 s). **e** UV light rewriting (64 mW/cm^2^ for 500 s). **f**–**h** EPSC curves corresponds to the pixel of the first row and column of the transistor array. The gray scales are the normalized current change. A constant source-drain voltage *V*_SD_ = 50 mV was applied to monitor the channel current.

Based on this long-term memory property, the smart sensing and image memorization of the letter V was realized using a 3 × 3 array consisting of VO_2_ transistors (Fig. 2). Laser light with the wavelengths of 650 nm and 375 nm at an intensity of 64 mW/cm^2^ were used to write this letter. Supplementary Figure 8 shows the simplified schematic of the illumination pattern. The changes of channel current (Δ*I*_D_) were normalized to 0-1 for the initial input signal and expressed by the shade of color. The images of letter V were all successfully input into the synapse array after 500 s exposure duration using the two light sources. The overall color of the letter written using the red light was significantly lighter than that of the letter written using UV light, indicating the small Δ*I*_D_ obtained under red light illumination. After removing the light stimuli, the Δ*I*_D_ of the array excited by red light almost disappeared after 500 s, while the Δ*I*_D_ stimulated by UV light decreased slightly at 1000 s and remained unchanged at 2500 s. This phenomenon indicates that the VO_2_-based neuromorphic synapse array can store UV information selectively. In order to demonstrate the erasing/writing operations in a more intuitive way, we erased the letter using a voltage pulse (−2V for a duration of 100 s), and rewritten it using UV light under the same conditions. The result shows that after erasing, the channel current almost returned to its initial state and remained stable for the next 500 s. The Δ*I*_D_ of the letter V after repeated writing was almost the same as the previous time. The above processes suggest that the VO_2_-based neuromorphic sensor array has excellent image memory capability and visible-blindness feature for the non-volatile change.

### Photo-induced non-volatile phase transition

**Fig. 3:**
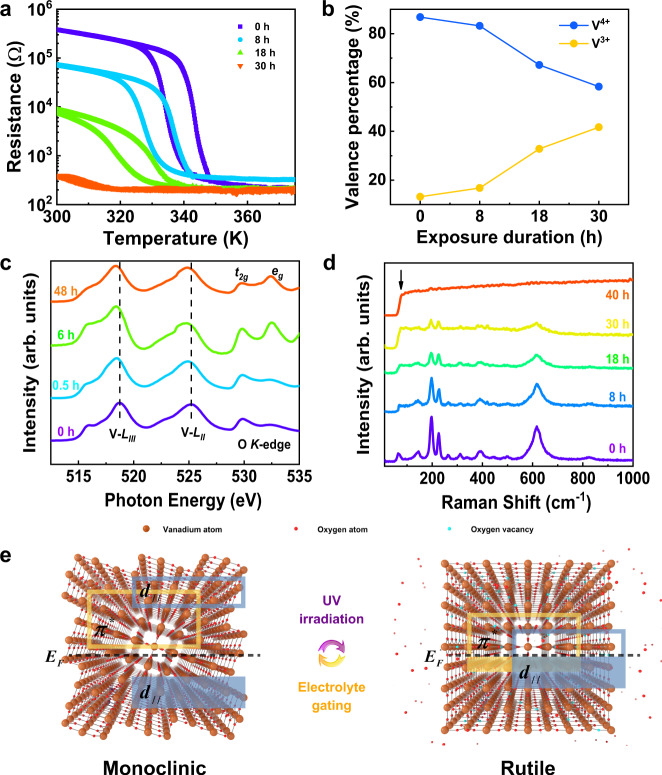
Photo-induced non-volatile phase transition. **a** Temperature-dependent resistance in VO_2_ films at various exposure durations of UV light at 84 mW/cm^2^. **b** Integrated area percentage of vanadium valence peaks at different UV irradiation duration. **c** V *L*-edge and O *K*-edge XAS curves for VO_2_ films at different UV irradiation durations (intensity of 100 mW/cm^2^). **d** Raman spectra at different UV irradiation durations (84 mW/cm^2^). **e** Schematic diagram of the reversible non-volatile phase transition driven by optical and electrolyte gating.

Next, we studied the underlying mechanism of selective memory property of VO_2_ at different wavelengths. The volatile response to visible light can be explained as the rapid recombination of photo-generated electron-hole pairs, while the non-volatile response to UV light could be ascribed to a photo-induced phase transition. To investigate the effects of UV light, we studied the temperature dependence of resistance at various UV light exposure durations (Fig. 3a). The as-grown VO_2_ film showed a typical phase transition, with resistance changing by three orders of magnitude. After UV light irradiation, the value of resistance in the low-temperature insulating phase gradually decreased, and was comparable to that of the metallic state after 30 h exposure. Moreover, we examined the response of channel current to UV light in different atmospheres (Supplementary Fig. 9). It was found that the *I*_D_ had a wide range of changes and good retention characteristic after irradiation in nitrogen and vacuum conditions. On the contrary, the current increase under an oxygen atmosphere was not obvious and subsided quickly after the light was removed. We can speculate that oxygen plays an important role in the optical control of phase transition, as is indicated by the difference in results obtained under oxygen-enriched and oxygen-deficient environment.

To further examine the stoichiometry changes inside the film qualitatively under UV light irradiation, we preformed X-ray photoelectron spectroscopy (XPS) on the VO_2_ films under the same light intensity and different exposure durations. The V 2*p* core-level peaks of the VO_2_ films (Supplementary Figure 10) showed that the V 2*p*_3/2_ peaks became gradually broader and shifted slightly toward the low binding energy direction after irradiation treatment. The V^3+^ state increased gradually in the film due to the increase of irradiation time, and so did the optically-induced oxygen vacancies. We fitted the V 2*p* core-level peaks with the parameters of V^4+^ 2*p*_3/2_ ≈ 516.4 eV, V^3+^ 2*p*_3/2_ ≈ 515.5 eV^37^. The continuous increased in proportion of the V^3+^ peak is evident. Based on the area percentage of different vanadium valence states in the V 2*p* spectrum, we roughly estimated their content and overlaid the calculation results on Fig. 3b. Before exposure to UV light, a small amount of V^3+^ signal was detected in the film, while the signal increased to ≈41.7% after 30 h of irradiation. This shows that a chemical environment of ~28.5% vanadium cations changed during this process. The concentration of oxygen vacancies was estimated as 4.85 × 10^21^ cm^−3^, which corresponds to a free electron concentration of 9.7 × 10^21^ cm^−3^. The change from V^4+^ to a lower valence state was also verified using X-ray absorption spectroscopy (XAS) of the V–*L* edge (Fig. 3c). The intensity ratio of the *t*_2g_ and *e*_g_ peaks in the O *K*-edge spectrum decreased substantially, indicating that the *d*_*//*_ and π* orbitals were gradually filled by electrons during UV irradiation. These results indicate that such optically-induced oxygen vacancies cause an electronic phase transition and suppress MIT behavior.

A Raman scattering experiment was employed to determine whether the UV light irradiation process is accompanied by structural phase transition in the VO_2_ film. The as-grown VO_2_ film exhibited typical M_1_ phase characteristics, with Raman peaks at 146, 198 (A_*g*_), 226 (A_*g*_), 262 (B_*g*_), 312 (A_*g*_), 339 (A_*g*_), 390 (A_*g*_), 443 (B_*g*_), 499 (B_*g*_), 617 (A_*g*_), and 827 cm^-1^ (B_*g*_)^38,39^ (Fig. 3d). As the exposure duration increased, metallic domains were gradually formed in the film, which was reflected in the Raman spectra as a sharp rise in the luminescence background (position indicated by arrow)^39,40^. Although the films mainly maintain the M_1_ phase under the irradiation durations of less than 40 h, the intensity of the characteristic peak of this phase significantly weakened. Finally, after an exposure duration of 40 h, a broad band between 200 and 1,000 cm^-1^ appeared in the spectra, proving that the VO_2_ structure was completely transformed from M_1_ phase to the R phase. The M_1_ phase portion was estimated from the Raman results as a function of exposure duration (Supplementary Fig. 11). The results show that along with the electronic structure phase transition, a structural phase transition also appeared during the optical control process.

Then we discussed the physical mechanism of VO_2_ neuromorphic sensor as shown in Fig. 3e. Since the activation energy for creating oxygen vacancies was calculated to be between 3 and 3.5 eV^35^, 375 nm UV light with a photon energy of 3.35 eV should be capable to release oxygen from the VO_2_ film under an oxygen-deficient environment to create oxygen vacancies in the crystal lattice. Red and green light cannot release the oxygen from the lattice, since their photon energies are lower than the activation energy of oxygen vacancy^41,42^, regardless of their light intensities. With the appearance of oxygen vacancies, the V atoms lose a few electrons and release them to the neighboring V-3*d* states; these electrons partially occupying the *d*_*//*_ and π* orbitals, leading to an electronic phase transition. Moreover, the oxygen vacancies in lattice and the differences in V ionic radius caused by the electrons’ release also lead to a strain in VO_2_, which transforms it from a low-symmetry monoclinic phase to a high-symmetry rutile phase and further induced the metallic phase^43^. This structural phase transition was further confirmed through the XRD pattern shown in Supplementary Fig. 12 and the related Supplementary Note 2. During the reset process, electrolyte gating could insert the oxygen ions back into the crystal lattice under a negative voltage^44^. With the decrease of oxygen vacancies in the channel, the VO_2_ structure gradually returns to its initial insulating monoclinic phase. In this manner, a reversible phase transition is achieved at room temperature through optical programing and electrical erasing. Since the transformation process is a photo-induced non-volatile phase transition and the metallic phase proportion increases almost linearly with the irradiation dosage, the device conductance shows good retention and linear dependency.

### Device performance on silicon wafer

We deposited VO_2_ film on a two-inch SiO_2_/Si wafer by magnetron sputtering technique, to further prove its silicon compatible potential. In order to study the structure of VO_2_ sputtered on Si substrates, we carried out a series of characterization experiments (Supplementary Fig. 13). The temperature dependence of resistance exhibits a significant change in 3 orders of magnitude, indicating that sputtered VO_2_ also has a typical phase transition characteristic. The phase composition is analyzed by powder X-ray diffraction and Raman spectroscopy. The film exhibits polycrystalline properties, mainly containing strong VO_2_ (011)_M1_ family peaks (space group P2_1_/c) and a weak ($\bar{4}02$)_M2_ peak (space group: C_2_/m). This result can be further verified by Raman spectrum. The sputtered VO_2_ film exhibits strong M_1_ phase characteristics, and is accompanied by weak M_2_ phase (131.09 cm^−1^) and A phase (966.88 cm^−1^) peaks^45^. The VO_2_ film grown by PLD is pure M_1_ phase, and it is found that the photo-induced phase transition is caused by the transition from M_1_ phase to R phase. Although the films grown using two methods have some differences (for example, temperature window and crystal orientation), the VO_2_ film sputtered on Si substrates dominated by the M_1_ phase also exhibits UV photo-induced phase transition similar to the VO_2_ epitaxial film grown on Al_2_O_3_ substrates.

**Fig. 4:**
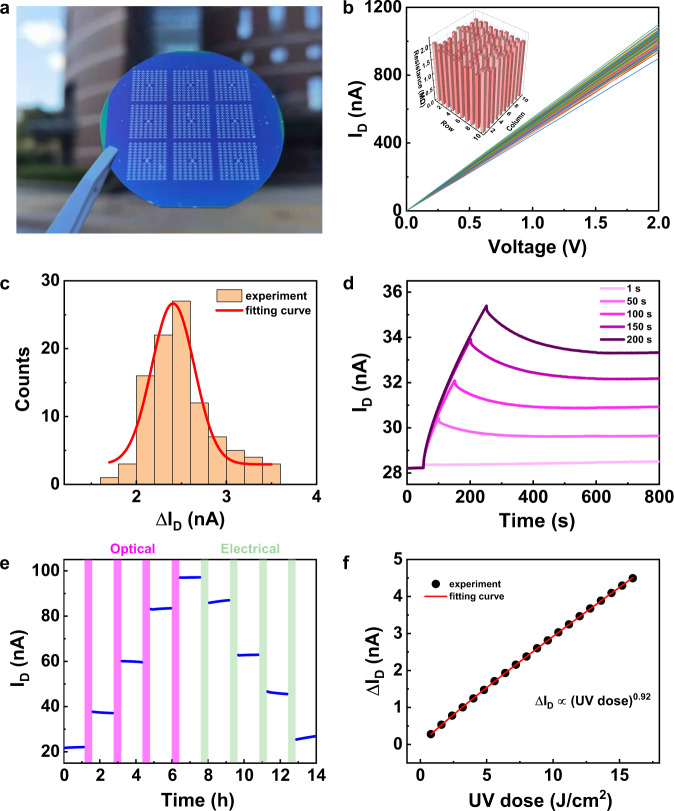
Device performance for VO_2_ film on silicon wafer. **a** Photograph of the ch wafer. **b** *I*–*V* curves of 100 devices selected from the array. Inset: histogram of the devices’ resistance. **c** Histogram of the photo response after 100 s of UV irradiation (84 mW/cm^2^). The red line is the fitting curve of a Gauss function. **d** spike-duration-dependent plasticity (intensity of 84 mW/cm^2^) of a selected device. **e** Multi-state retention properties of VO_2_ devices. The potentiation states were produced via UV irradiation, while the depression states via electrolyte gating. The regulation time was 0.5 h and the retention time was 4000 s. **f** Relationship between *I*_D_ and incident UV dose with data extracted from Supplementary Fig. 15. The red line is the fitting curve of a power function. A constant source-drain voltage *V*_SD_ = 50 mV was applied to monitor the channel current.

Then, a 3 × 3 device array was fabricated with the same device structure as prepared on r-Al_2_O_3_, each array having 103 devices (Fig. 4a). We conducted the same optical writing operations to verify the photo-induced phase transition characteristics of silicon-based devices. We randomly selected 100 devices from the arrays, and examined their channel resistance and response to UV light (Fig. 4b, c). The *I*–*V* curves distribution of the devices are relatively concentrated, and the resistance histogram (Fig. 4b inset) shows that the overall device resistance on the Si wafer is ~2 MΩ, which reflects the uniformity of the film growth. The histogram of the statistical distribution of the photo response shows that after 100 s of UV irradiation at 84 mW/cm^2^, 96% of the devices have a channel current change of more than 2 nA. The fitting results show that the distribution of Δ*I*_D_ was a normal distribution. The selective memorization tested under different wavelengths of light showed that the silicon-based device also had the non-volatile memory characteristics for UV light only (Supplementary Fig. 14). In addition, the changes of channel currents were tested against UV exposure duration and UV light intensity as depicted in Fig. 4d and Supplementary Fig. 14, respectively. The results show that the multi-level memory feature of the device can be adjusted by controlling the UV irradiation conditions. Moreover, we carried out the optical programming and electrical erasing operations on the transistors (Fig. 4e), which showed reversibility and retention characteristics. To further characterize its non-volatile multi-level features, a series of UV light pulses (intensity of 84 mW/cm^2^, duration of 10 s) were used to program the device (Supplementary Fig. 15). Throughout the writing process, *I*_D_ showed LTP synaptic plasticity and the channel current exhibited almost the same response to each UV pulse. We extracted the accumulation of Δ*I*_D_ and plotted it in Fig. 4f along with the UV dose, which can be calculated by the following equation: UV dose (mJ/cm²) = UV Intensity (mW/cm²) × Exposure Time (s). The curve of Δ*I*_D_ dependence of the UV dose is fitted well using a power function with a power of 0.92. The above results indicate that the VO_2_ grown on SiO_2_/Si wafer has the same perception and storage characteristics of UV light. The wafer-scale integration capability of VO_2_ lays a good foundation for future applications of neuromorphic UV sensors (Supplementary Table 2).

Furthermore, we investigated the effect of device size on the non-volatile channel current change under the same UV dose. Considering the effect of UV radiation on the channel in the out-of-plane direction, the channel conductance *G* can be described as: $G=\frac{W}{L}{\int }_{0}^{H}\sigma \left(h\right){dh}$, where *W*, *H*, *L* are the width, thickness, and length of the channel, respectively. *σ*(*h*) is the conductivity of VO_2_ channel, which is the function of the depth. The integration part is named as *σ*_s_. The change of *σ*_s_ is related to the concentration of the induced oxygen vacancies determined by UV dose, and is independent on the lateral size of the device. The change in channel current Δ*I*_D_ can be represented by $\triangle {I}_{D}={{V}_{D}\triangle \sigma }_{S}\frac{W}{L}$. It can be seen that Δ*I*_D_ is independent of the device area. Besides, VO_2_ films down to nanoscale still have the phase transition characteristics^28,46,47^. Therefore, scaling down will not affect UV neuromorphic characteristics of the device. In addition, the device performance can be further improved by increasing the ratio of *W*/*L*.

### Image preprocessing and recognition

**Fig. 5:**
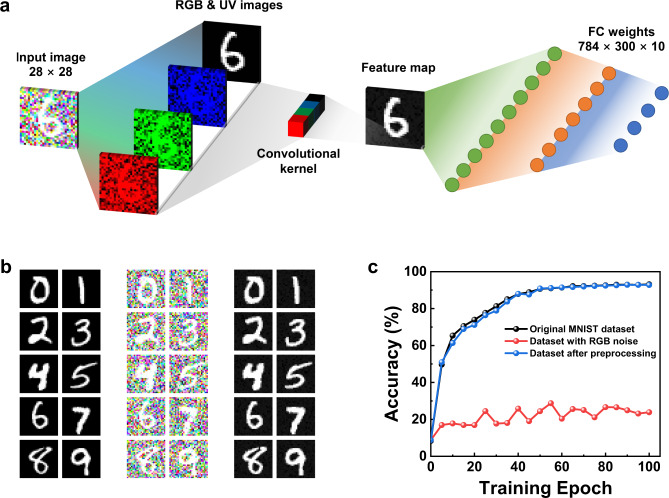
Image recognition using the VO_2_-based UV neuromorphic sensor system. **a** Realization of neuromorphic preprocessing function to achieve image noise reduction utilizing the sensor array. The system can be spatially divided into a convolution kernel array part for visual information preprocessing and an ANN part for image recognition. **b** Three types of pre-prepared images including the original MNIST test images (left columns), specially processed images with R, G and B Gaussian noise (middle columns), and the images after noise reduction by the sensor array (right columns). **c** Recognition accuracy with and without neuromorphic preprocessing.

At present, most machine processing of visual information is in the light range which is visible to human beings^48^. This is because the visible light information is one of the main types of external information that guides human life. However, non-visible light also contains much important visual information, and this type of information plays an important role in guiding the behavior of creatures whose perceptible light range is different from humans’^11^. For example, the significant absorption of UV by nectar causes the stamen to be obviously darker than the petals in the UV range, and the perceptible light range of bees includes this UV part of the spectrum, which is imperceptible by human^3^. This characteristic UV information of nectar could help bees find the target flowers quickly during nectar collection. In addition, it is worth noting that when people try to identify certain characteristic information, redundant information will be automatically filtered out by the receptor, just like when people focus on a specific color, the rest of the color information will be mostly filtered out by their eyes. Such information extraction behavior can be defined by designing a suitable convolution kernel, which is a matrix of weight values used to perform a weighted average operation on pixels in a small area^28,49^. Since the proposed VO_2_ device shows a difference in its UV and visible light response, it can be used to simulate the behavior of bees focusing on UV information during nectar collection. A UV visual system with preprocessing (i.e. extraction of UV characteristic information) and recognition functions was modeled using computer simulation. The schematic diagram of its operation is shown in Fig. 5a. Based on the different functions implemented, the visual system was spatially divided into a convolution kernel array part for visual information preprocessing and an ANN part for image recognition after preprocessing.

To demonstrate the difference in image recognition with or without the ability to focus on UV information, the standard MNIST handwritten digital images (at a size of 28 × 28 pixels each) were used. An additional value independent of the RGB values was added in the computer simulation to introduce the invisible UV information into traditional RGB images. Based on the different responses of the device to 650 nm, 532 nm, 450 nm, and 375 nm light, each VO_2_ UV visual sensor was formed as a convolution kernel with a size of 1 × 1 × 4. Such convolution kernel performs weighted average processing on the four-color values (RGB and UV values) of a single pixel. After convolution, the resulting feature map reflected the scene that bees can observe when collecting nectar (i.e. an image with more abundant UV information and scarce visible light information). Subsequently, the preprocessed image was input into a fully connected (FC) ANN for recognition, which included an input layer (784 neurons), a hidden layer (300 neurons), and an output layer (10 neurons). The detailed operation mechanisms of the convolution kernel and the ANN are described in Supplementary Note 3.

For image recognition, the ANN part was firstly trained using the back-propagation algorithm and 60,000 images from the MNIST train dataset. Subsequently, three types of test datasets were fed into the ANN to compare the differences in image recognition accuracy under different conditions. These included the original MNIST test dataset, the same dataset with blurred visible light information, and the dataset after preprocessing, as shown in Fig. 5b. The second type of dataset was obtained by adding RGB Gaussian noise to the first dataset. Figure 5c shows the dependence of the recognition accuracy for these three datasets on the training epoch number. It can be seen that the recognition accuracy for images containing RGB Gaussian noise only reached about 24%, which was only slightly higher than the initial accuracy. This means that in this case, the recognition system can hardly recognize the characteristic information. In contrast, after the device preprocesses the UV information, the recognition accuracy of the image reached about 93%, which was the same as that obtained for the original MNIST. This result shows the effectiveness of the device in extracting ultraviolet information. In addition, this phenomenon was consistent with the fact that bees can identify which flower has nectar accurately, while humans cannot achieve this from visual information alone. Moreover, previous studies showed that the photoelectric response speed of VO_2_ is in the sub-picosecond scale^50,51^, ensuring its potential application in real-time monitoring systems.

## Discussion

In summary, we have successfully fabricated and demonstrated a VO_2_ optoelectronic synapse able to perceive and memorize UV light stimuli due to its photo-induced non-volatile phase transition. Benefitting from a phase conversion ratio linearly related to the light dosage, the device has linear writing and retention behaviors. Electrolyte gating was utilized as the electrical erasing process. Moreover, we fabricated a wafer-scale integrated neuromorphic sensor array and proved that the VO_2_ film on the silicon wafer also achieves optical control of phase transition, indicating the possibility of commercial mass production of neuromorphic sensors. In terms of high-density integration, it is worthy to further study the photo-induced phenomena of oxide materials with high phase transition temperature^52^. An ANN was simulated for the recognition of handwritten digit images from the MNIST dataset after the addition of random Gaussian noise. The results demonstrate that using the neuromorphic preprocessing process to reduce redundant data, the image recognition rate increased from 24% to 93%. Our work shows that VO_2_ has photo-induced non-volatile phase transition property and large-scale integration potential, which lays the foundation for the practical applications of neuromorphic sensor devices.

## Methods

### Sample preparation

The 20 nm VO_2_ thin films were epitaxially grown on r-plane ($1\bar{1}02$) Al_2_O_3_ substrates, using the pulsed laser deposition with a 308-nm XeCl excimer laser, an energy density of about 1 J/cm^2^ and a repletion rate of 3 Hz. The VO_2_/Al_2_O_3_ films were deposited at 485 °C in a flowing oxygen atmosphere with pressure 1.0 Pa. The samples were cooled down to room temperature at 20 °C/min. The deposition rate of VO_2_ films was calibrated by X-ray Reflection.

The wafer-scale VO_2_ film with a thickness of 20 nm was deposited on 2-inch SiO_2_ (300 nm)/Si wafer by RF magnetron sputtering using V_2_O_5_ target. It was performed with RF power of 150 W with a flow of 70 sccm Ar and working pressure of 7 mTorr. The oxygen content in the as-grown film was well-controlled after an annealing at 650 °C for 0.5 h under vacuum condition.

### Device fabrication

The thin films were patterned into channels with a coplanar gate structure using standard photolithography and argon-ion etching. The effective device area is 50 µm × 180 µm. The length between the gate electrode and channel is 10 µm. The 70 nm Pt layer was deposited as electrodes by RF sputtering. The transistor device was completed by dropping an ionic liquid N, N-diethyl-N-(2-methoxyethyl)-N-methylammoniumbis-(trifluoromethylsulphonyl)-imide (DEME-TFSI) on the channel and gate electrodes.

### Material characterization

X-ray diffraction patterns of the VO_2_ film was performed using a Rigaku SmartLab instrument with a 2*θ* range from 20 to 45° in step of 0.05°. XPS measurements were performed on ThermoFisher Scientific ESCALAB 250X under monochromatic Al Kα radiation with an energy of 1486.6 eV. XAS measurements were performed on via total electron yield method, and the background vacuum level was 6 × 10^−7^ Torr. Raman spectrum was analyzed using the alpha300 R microscope under 532 nm laser excitation. Powder X-ray diffraction pattern of the VO_2_ films sputtered on Si substrates was measured using a Rigaku Ultima IV instrument with a 2*θ* range from 20 to 60°.

### Device characterization

All the electrical characterizations were measured in a Lakeshore probe station with a Keithley 4200 semiconductor parameter analyzer in vacuum at room temperature. An UV laser with a wavelength of 375 nm were used for the optical switching in the experiment.

## Supplementary information

Supplementary Information

## Data Availability

All relevant data are available within the Article and Supplementary Information, or available from the corresponding authors upon reasonable request.

## Code availability

All code used in simulations supporting this article is available from the corresponding authors upon reasonable request.
