# Supplementary material for: Photo-induced non-volatile VO2 phase transition for neuromorphic ultraviolet sensors
Source: Nat Commun. 2022 Apr 1;13:1729. doi: 10.1038/s41467-022-29456-5 (PMC8975822; doi:10.1038/s41467-022-29456-5)
Supplement: Supplementary file 1 — Supplementary Information [file 41467_2022_29456_MOESM1_ESM.pdf]

Supplementary Information

Photo-induced non-volatile VO_2 phase transition for neuromorphic ultraviolet sensors

Ge Li[1,2,#], Donggang Xie[1,3,#], Hai Zhong[1], Ziye Zhang[1,4], Xingke Fu[1], Qingli Zhou[4], Qiang Li[5], Hao Ni[3], Jiaou Wang[6], Er-jia Guo[1], Meng He[1], Can Wang[1,2], Guozhen Yang[1], Kuijuan Jin[1,2,*], and Chen Ge[1,2,*]

[1] Beijing National Laboratory for Condensed Matter Physics, Institute of Physics, Chinese Academy of Sciences, Beijing 100190, China

[2] University of Chinese Academy of Sciences, Beijing 100049, China

[3] College of Science, China University of Petroleum (East China), Qingdao, 266580, China

[4] Key Laboratory of Terahertz Optoelectronics, Ministry of Education, and Beijing Advanced Innovation Center for Imaging Theory and Technology, Department of Physics, Capital Normal University, Beijing 100048, China

[5] College of Physics, University-Industry Joint Center for Ocean Observation and Broadband Communication, State Key Laboratory of Bio-Fibers and Eco-Textiles Qingdao University, Qingdao 266071, China

[6] Beijing Synchrotron Radiation Facility, Institute of High Energy Physics, Chinese Academy of Sciences, Beijing 100049, China

[#] These authors contributed equally: Ge Li, Donggang Xie

*Correspondence and requests for materials should be addressed to K.J. (email: kjjin@iphy.ac.cn) or to C.G. (email: gechen@iphy.ac.cn).

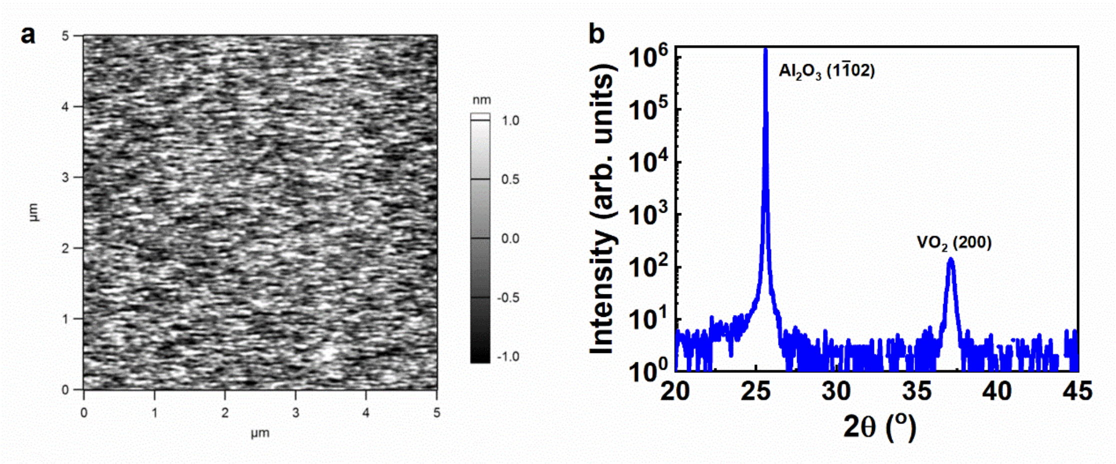

Supplementary Figure 1. Sample characterization of VO_2 films. a The AFM profile of 20 nm VO_2 film on r-Al_2O_3. The surface roughness is 536 pm. **b** Symmetrical XRD θ-2θ patterns for VO_2 epitaxial thin films ($20^o \leq 2\theta \leq 45^o$).

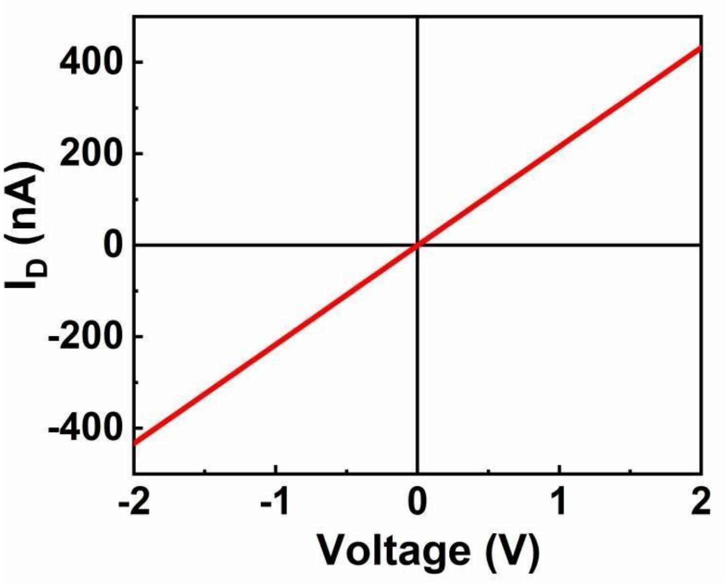

Supplementary Figure 2. Electrical property of VO_2 channel. The current-voltage (I-V) curve between the source and drain electrodes. The result implies good ohmic contact.

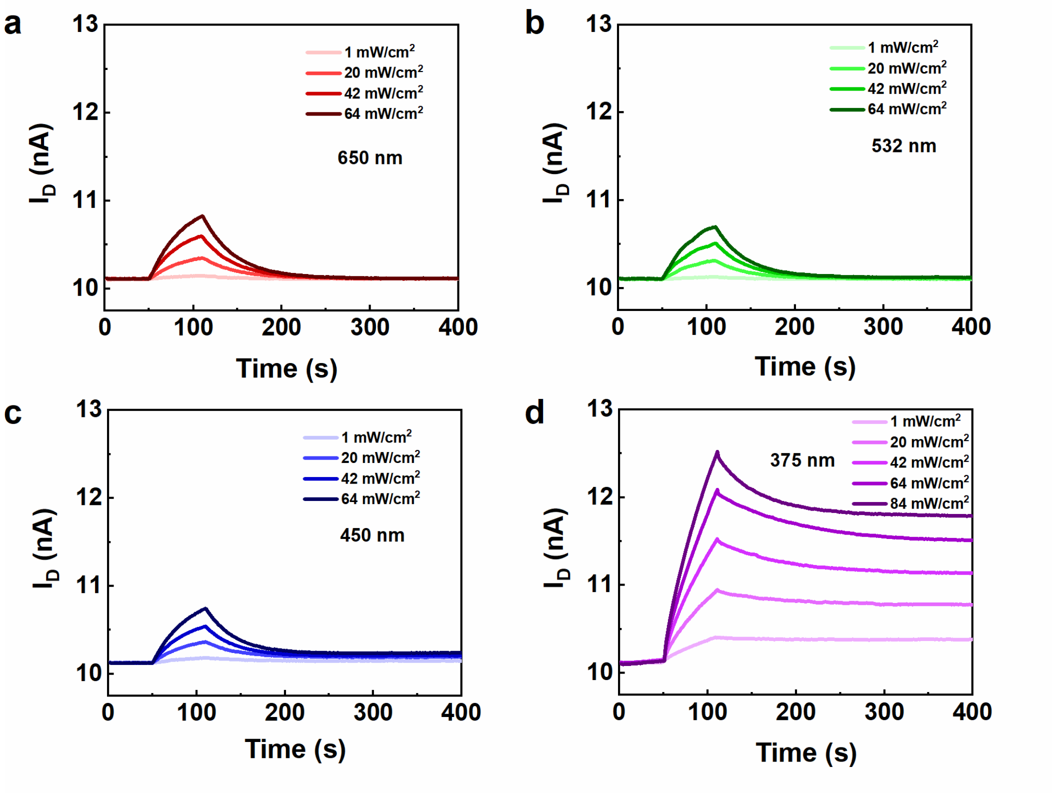

Supplementary Figure 3. The effect of the light exposure on the channel current at different wavelengths. EPSC under various light intensity of **a** red light, **b** green light, **c** blue light, and **d** UV light (the duration of 60 s).

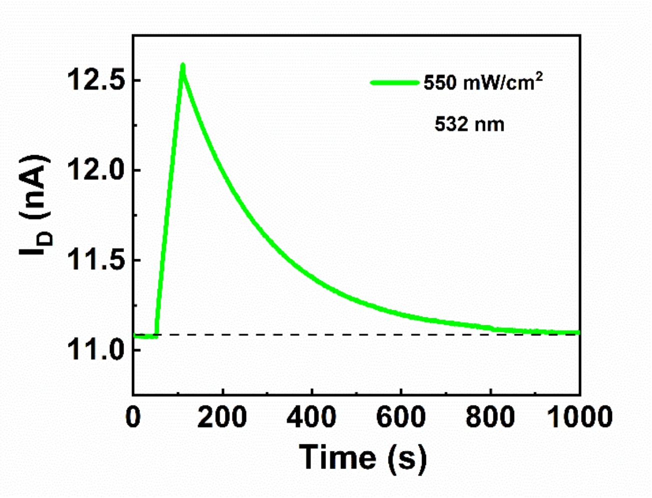

Supplementary Figure 4. The effect of strong green laser irradiation. EPSC under 550 mW/cm^2 light intensity of green light.

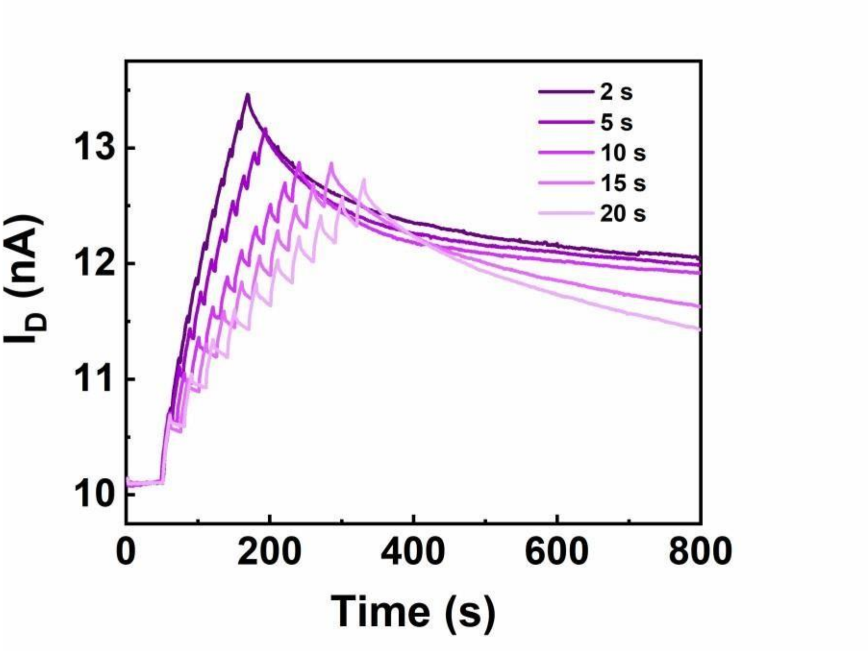

Supplementary Figure 5. Spike-frequency-dependent plasticity. The EPSCs are measured under UV irradiation with different interval duration (light intensity of 84 mW/cm^2, the duration of 10 s).

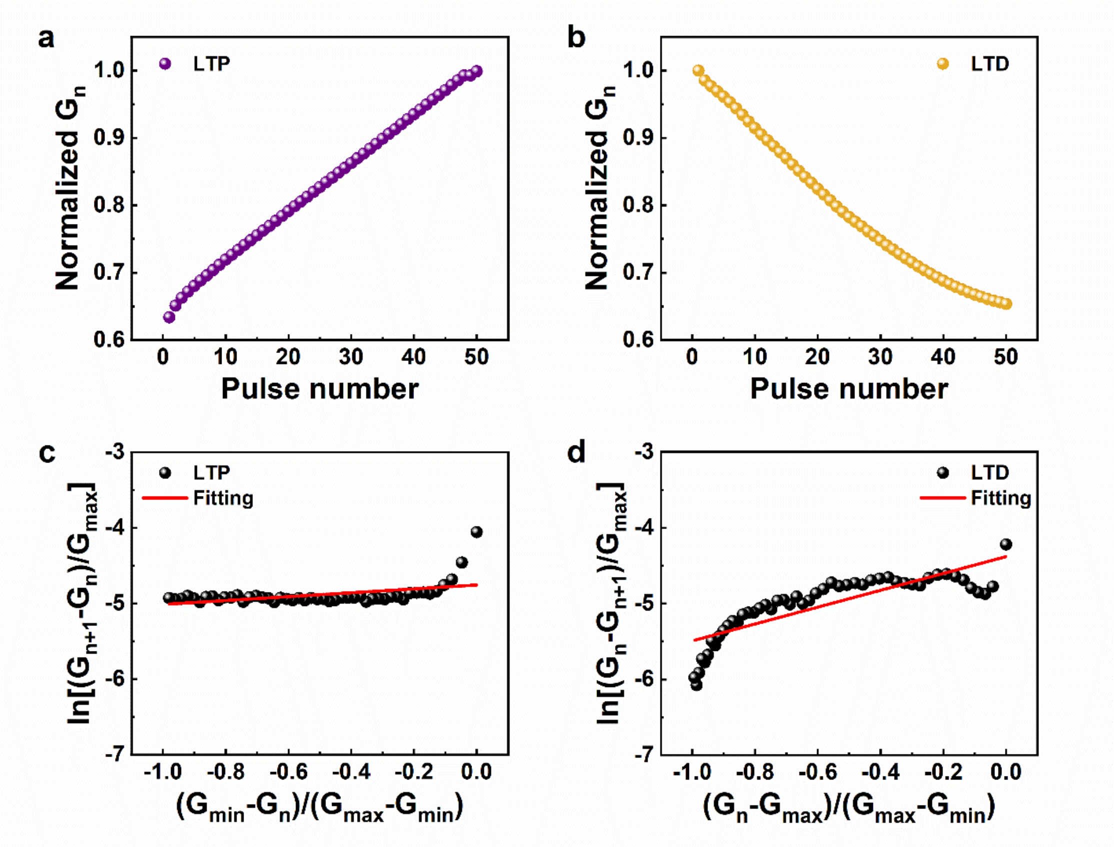

Supplementary Figure 6. The original and processed experimental LTP/LTD data. **a** The LTP experimental results after normalized the conductance. **b** The LTD experimental results after normalized the conductance. **c** The processed experimental LTP data and its fitting curve. **d** The processed experimental LTD data and the corresponding fitting curve.

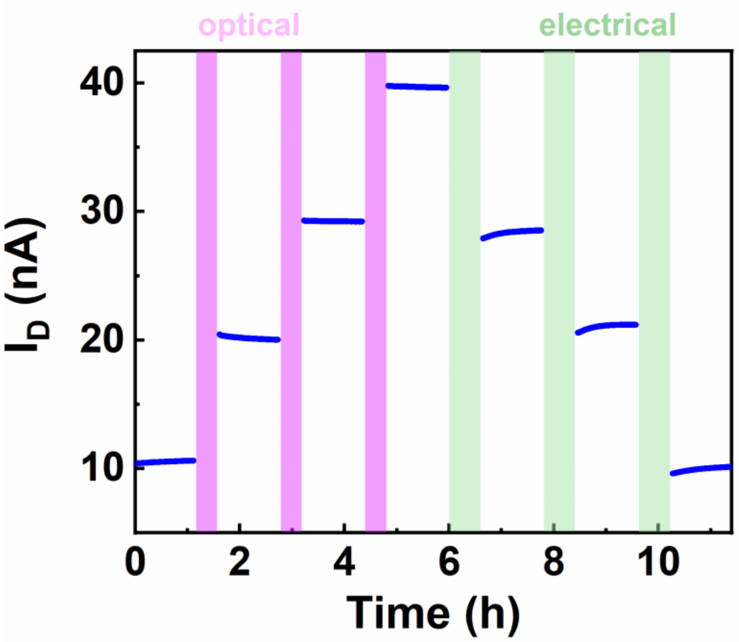

Supplementary Figure 7. Multi-state retention properties of VO_2/r-Al_2O_3 devices. The potentiation states are produced by UV irradiation for 0.5 h pulses spaced 4,000 s apart, and the depression states are produced by electrolyte gating for 1h pulses spaced 4,000 s apart.

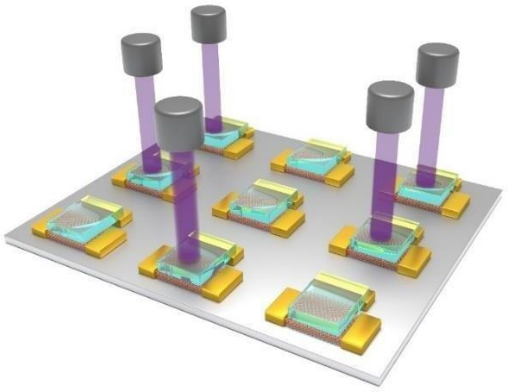

Supplementary Figure 8. A simplified schematic of the illumination pattern. The schematic shows our 3 × 3 device array and the experimental programming method for this array.

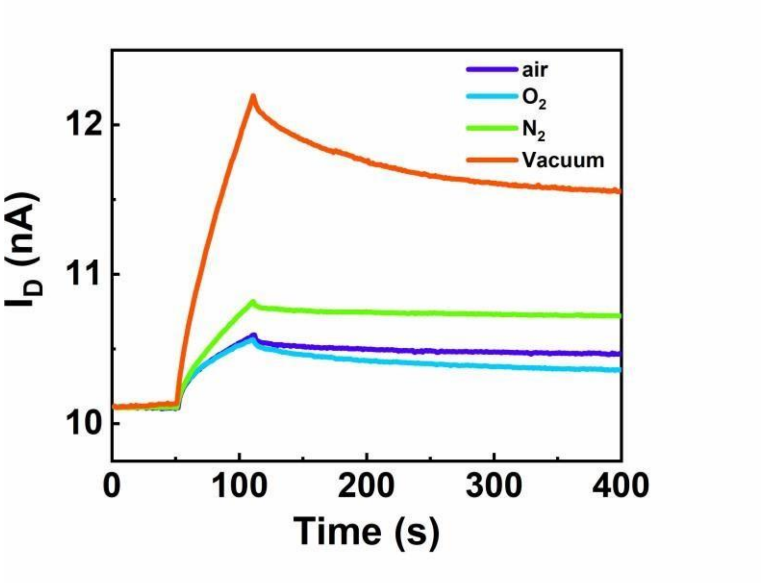

Supplementary Figure 9. The influence of atmosphere on the change of channel current. EPSC of VO_2 device irradiated by UV light in different atmospheres (light intensity of 84 mW/cm^2, the duration of 60 s).

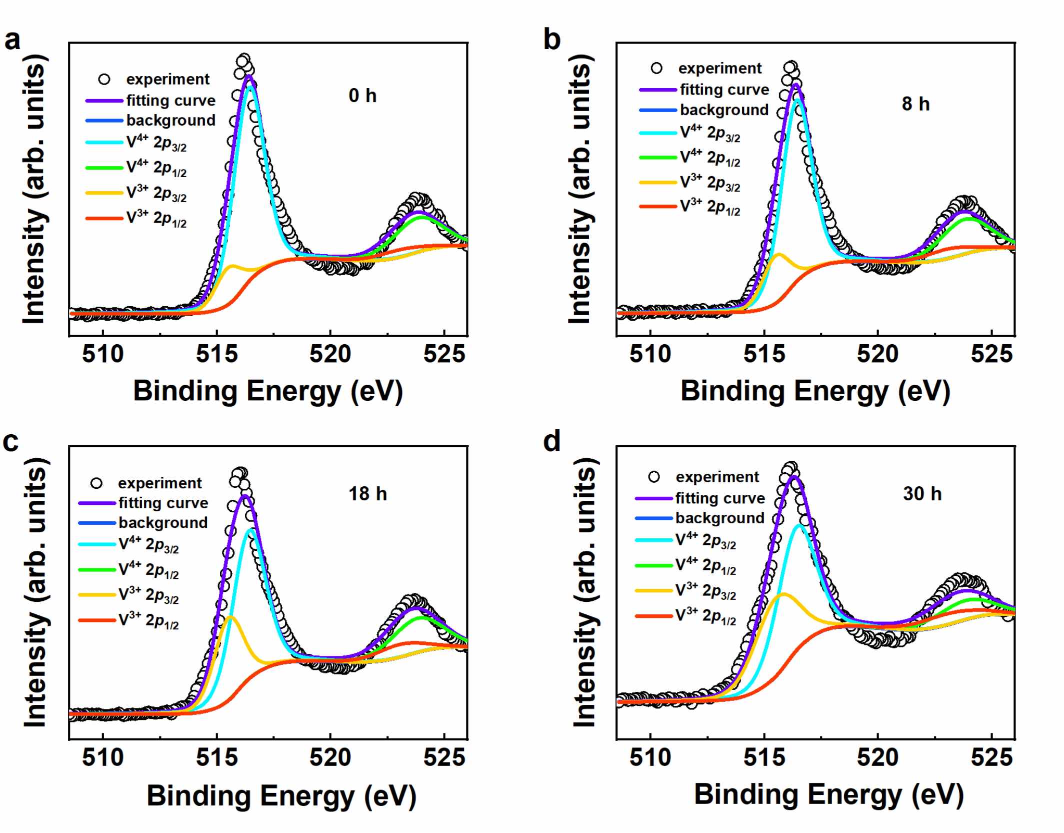

Supplementary Figure 10. The XPS spectra measured around V 2p peaks. The samples were irradiated with UV light intensity of 84 mW/cm^2 for **a** 0 h, **b** 8 h, **c** 18 h, and **d** 30 h.

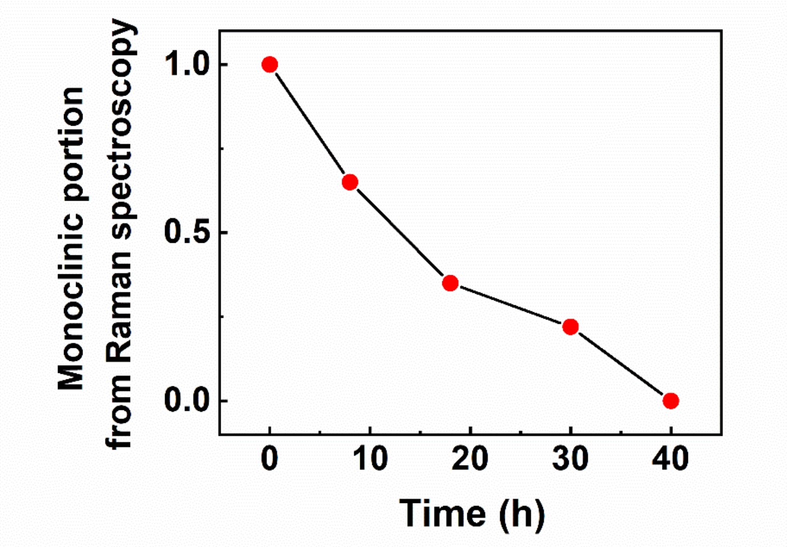

Supplementary Figure 11. Monoclinic portion as a function of UV exposure duration. The monoclinic portion is extracted from Fig. 3d.

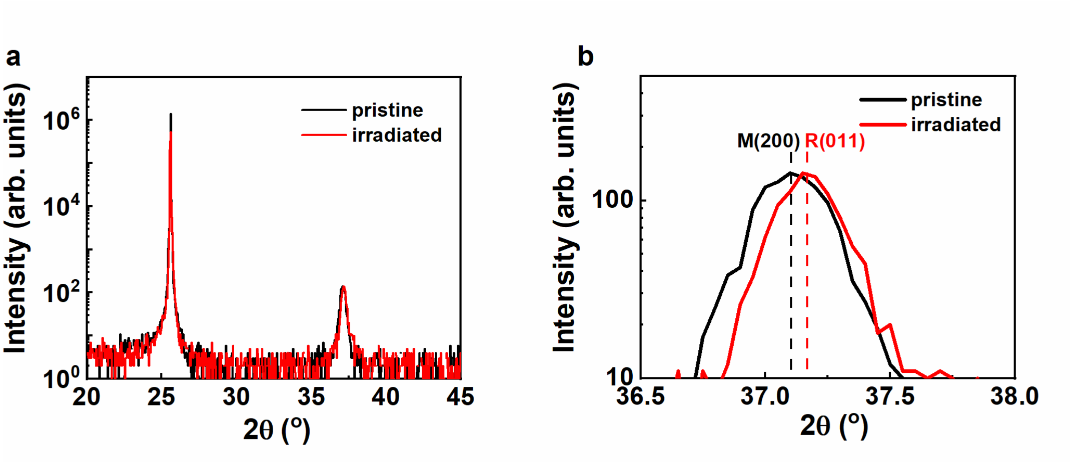

Supplementary Figure 12. Symmetrical XRD *θ*-2*θ* patterns. The XRD of VO_2 film measured before and after UV irradiation **a** (20° ≤ 2*θ* ≤ 45°); **b** (36.5° ≤ 2*θ* ≤ 38°).

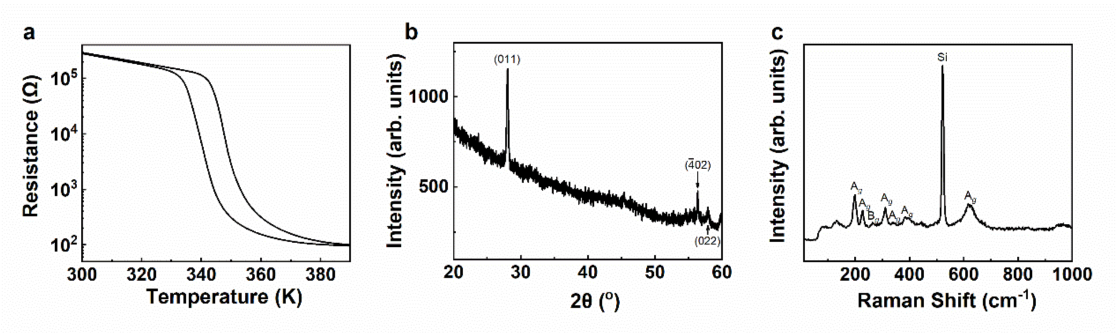

Supplementary Figure 13. Basic physical properties of VO_2 films sputtered on Si substrates. a Temperature-dependent resistance in VO_2 film. **b** Powder X-ray diffraction pattern of VO_2 film ($20^o < 2\theta < 60^o$). **c** Raman spectrum of VO_2 film.

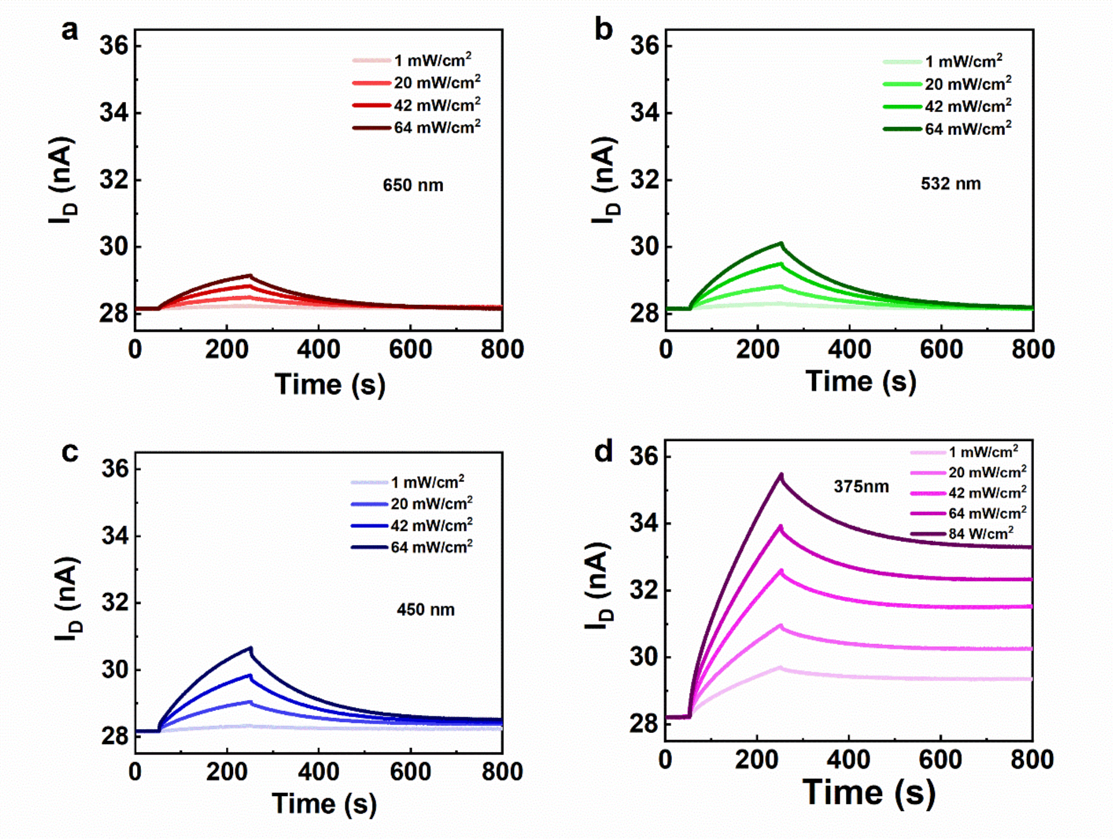

Supplementary Figure 14. The effect of the light exposure on the channel current at different wavelengths. EPSC under various light intensity of **a** red light, **b** green light, **c** blue light and **d** UV light (the duration of 200 s).

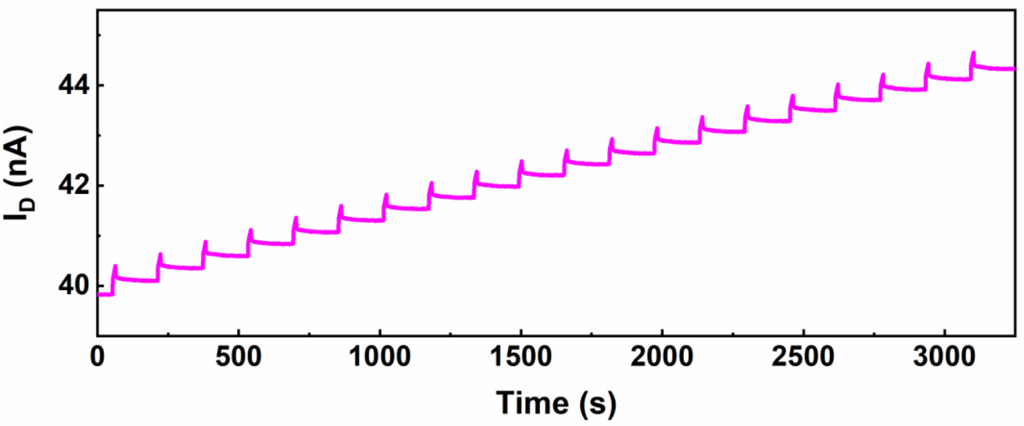

Supplementary Figure 15. The non-volatile multi-level conductance switching. LTP process achieved by applying 20 UV pulses at 84 mW/cm^2 for 10 s and a pulse interval of 150 s.

Supplementary Note 1

The non-linearity (NL) values for the potentiation and depression were calculated by fitting LTP/LTD profiles (Supplementary Figure 6) using the following relations:

$$G_{\mathrm{n+1}} = G_{\mathrm{n}} + \Delta G_{\mathrm{P}} = G_{\mathrm{n}} + \alpha_{\mathrm{P}} e^{-\beta_{\mathrm{P}} \frac{G_{\mathrm{n}} - G_{\mathrm{min}}}{G_{\mathrm{max}} - G_{\mathrm{min}}}} \; (\Delta G^{+} or \Delta G^{-} > 0,\; G^{+} or G^{-} \uparrow)$$

Supplementary Equation 1,

$$G_{\mathrm{n+1}} = G_{\mathrm{n}} + \Delta G_{\mathrm{D}} = G_{\mathrm{n}} - \alpha_{\mathrm{D}} e^{-\beta_{\mathrm{D}} \frac{G_{\mathrm{max}} - G_{\mathrm{n}}}{G_{\mathrm{max}} - G_{\mathrm{min}}}} \; (\Delta G^{+} or \Delta G^{-} < 0,\; G^{+} or G^{-} \downarrow)$$

Supplementary Equation 2.

Here, G_{n} and $G_{\mathrm{n+1}}$ denote the conductance of nth and (n+1)th pulses. G_{min} and G_{max} are the minimum and maximum conductance. α_{P} and α_{D} are the differences in the conductance between two points on the potentiation and depression curves. β_{P} and β_{D} denote the curvatures of the potentiation and depression curves (i.e., NL).

The fitting curve and extracted fitting parameters of the experimental LTP/LTD data are shown in Supplementary Figure 6 c-d and summarized in Supplementary Table 1, respectively.

Supplementary Note 2

The X-ray diffraction (XRD) pattern demonstrated that the structure of VO_2 transformed from the monoclinic phase to the rutile phase under UV exposure (Supplementary Figure 12). Due to the expansion of lattice during monoclinic distortion, the XRD peak of VO_2 shifted from 37.1^{o} to 37.15^{o}, corresponding to the Bragg angles of $(200)_{M}$ (2θ=37.088^{o}) and $(011)_{R}$ (2θ=37.12^{o}) in the XRD standard card, respectively. This result indicated that with the induced of oxygen vacancies in the crystal lattice, VO_2 underwent a structural phase transition from low-symmetry to high-symmetry.

Supplementary Note 3

Construction of convolution kernel

In convolution processing, the weighted average of pixels in a small area in the input image becomes the corresponding pixel in the output image. The weight value is defined by a function, which is called convolution kernel. Convolution kernels are commonly used in image processing applications. For example, the Sobel operator, which is attributed to filtering operations, is also one of the current convolution kernels. Previous study [1] showed that the convolution kernel in the convolutional neural network will have the function of local feature extraction after training. Here, we used the proposed device as a convolution kernel for extracting ultraviolet feature in the application of neural networks. It is worth noting that such convolution kernel is used to perform a weighted average operation on the RGB and UV values of a single pixel. Therefore, a device array with the same size as the input image can realize the whole convolution processing in one step.

The convolution kernel is essentially a weight function, so that a set of weight functions based on device characteristics need to be defined in order to be used in subsequent computer simulation. Since the device exhibits a unique wavelength-dependent light response, the result of convolution processing based on the device characteristics is the sum of the responses to light of different wavelengths. The equation describing the convolution operation is as follows:

$$f_{Output} = \frac{f_R(R) + f_G(G) + f_B(B) + f_{UV}(UV)}{4} = \Delta I_R + \Delta I_G + \Delta I_B + \Delta I_{UV} = \Delta I_{Output}$$

Supplementary Equation 3.

where f_{Output} is the output after convolution processing on a pixel in the input image, which is the output value obtained by weighting and averaging the RGB and UV values of a pixel. $f_R(R)$, $f_G(G)$, $f_B(B)$ and $f_{UV}(UV)$ are the weight functions of the R, G, B and UV values in the image, respectively. Corresponding to the device characteristics, the output is the sum of the current changes of the device in response to light at 650 nm, 532 nm, 450 nm, and 375 nm. Based on the experiment data of EPSC under various light intensity of red, green, blue, and UV light (Supplementary Figure 3), the relationship between the current response and

the light intensity under the irradiation of different colors of light can be obtained. It is assumed that the values of RGB and UV have a linear relationship with the light intensity the corresponding wavelength. The weight function of the convolution operation can be defined as the following four piecewise functions:

$$f_R(R) = 4\Delta I_R = \begin{cases} 0 & , \quad R = 0 \\ 0.02352 & , \quad 0 < R \leq 8.5 \\ -0.00752 & , \quad 8.5 < R \leq 85 \\ -0.00692 & , \quad 85 < R \leq 170 \\ -0.00968 & , \quad 170 < R \leq 255 \end{cases} \quad \text{Supplementary Equation 4,}$$

$$f_G(G) = 4\Delta I_G = \begin{cases} 0 & , \quad G = 0 \\ -0.04564 & , \quad 0 < G \leq 8.5 \\ 0.00456 & , \quad 8.5 < G \leq 85 \\ 0.02728 & , \quad 85 < G \leq 170 \\ 0.03592 & , \quad 170 < G \leq 255 \end{cases} \quad \text{Supplementary Equation 5,}$$

$$f_B(B) = 4\Delta I_B = \begin{cases} 0 & , \quad B = 0 \\ 0.04488 & , \quad 0 < B \leq 8.5 \\ 0.24236 & , \quad 8.5 < B \leq 85 \\ 0.32436 & , \quad 85 < B \leq 170 \\ 0.45616 & , \quad 170 < B \leq 255 \end{cases} \quad \text{Supplementary Equation 6,}$$

$$f_{UV}(UV) = 4\Delta I_{UV} = \begin{cases} 0 & , \quad UV = 0 \\ 1.03688 & , \quad 0 < UV \leq 8.5 \\ 2.62052 & , \quad 8.5 < UV \leq 85 \\ 4.06644 & , \quad 85 < UV \leq 170 \\ 5.55588 & , \quad 170 < UV \leq 255 \end{cases} \quad \text{Supplementary Equation 7.}$$

Substituting Supplementary Equation 4-7 into Supplementary Equation 3, the convolution kernel used to describe the device characteristics was constructed. Combining Supplementary Equation 4-7, it can be found that the weight function of UV value is much larger than that of RGB value. After weighted average, that is, convolution processing, the UV information in each pixel will occupy a dominant position, while the RGB information will be greatly suppressed.

The preparation of test dataset

To compare the differences of image recognition accuracy under different conditions, three test datasets were used, the first of which is the initial MNIST test dataset downloaded from the website (http://yann.lecun.com/exdb/mnist/). This type of test dataset was used to verify the reliability of the simulated ANN built based on the proposed devices. The test dataset includes 10,000 test images (where each image is 28 × 28 pixels). It is worth noting that the test images are all grayscale, that is, each pixel is represented by one value. In order to store information of different colors in the images, RGB mode had been used. However, RGB values could only store visible light information. Therefore, a separate value should be introduced to storage the UV information. Subsequently, in order to design a set of images with fuzzy visible light information and clear UV information (i.e. the characteristic information that humans cannot recognize, but bees can recognize), the Gaussian noise was attached to the RGB values and the values representing UV information were not changed, which formed the second test dataset. The third test dataset is the result obtained after the second group of data set was preprocessed by the simulated convolution kernel array. In the third test dataset, the UV information is strengthened and the visible light information is weakened, which simulates the situation that bees can perceive and focus on the UV information when collecting nectar.

The architecture of ANN

The simulated three-layer ANN includes an input layer (784), a hidden layer (300), and an output layer (10). The activation functions of the hidden layer and output layer were Relu and Softmax, respectively. It is worth noting that the synaptic weight, in practical applications, could have both positive and negative values, while the conductance of the

proposed device was always positive. Therefore, each synaptic weight (w) in the ANN were determined by a pair of normalized conductance values, i.e., $w = (G^{+} - G^{-})/G_{\text{max}}$.

Weight update method

For the weight updating, the back-propagation algorithm was used for training process. The weight update rule had been defined by using the experimental LTP/LTD data. The long-term potentiation (LTP) and long-term depression (LTD) were achieved by illuminating optical pulses of 375 nm wavelength and applying electrical pulses variety from -1.5V to -3.5V, respectively.

Next, the sign of weight change was calculated based on the output value and label value to determine whether the synaptic weight needed to increase (potentiation) or decrease (depression). In addition, to prevent overfitting, if $|\Delta w| < 5 \times 10^{-4}$, the conductance of *w* would not be updated, otherwise, the conductance would be updated as follows. Considering that at most one pulse was applied to each device update, there were three ways to update the device conductance, which were as follows: applying an optical pulse, applying an electrical pulse, and applying no pulse. Since the *w* was determined by the conductance of the paired devices, there were nine methods in total. Next, the optimal update method would be used to update the conductance.

Calculation of learning accuracy

After parameter updating, the three types of test datasets would be sent to the ANN for image recognition and the recognition accuracy of each dataset was calculated by:

$$Accuracy(\%) = \frac{n}{N} \times 100\% \quad \text{Supplementary Equation 3,}$$

where *Accuracy* denotes the recognition accuracy, *n* is the number of correctly identified images, and *N* is the total number of images in the test dataset, which was 10,000.

Normalized G_{max}	Normalized G_{min}	Fitting parameters STP			Fitting parameters STD		
		$\ln\alpha_P$	α_P	β_P	$\ln\alpha_D$	α_D	β_D
1	0.5640	-4.7556	0.0086	0.2566	-4.3797	0.0125	1.1115

Supplementary Table 1. The fitting parameters extracted from the experimental LTP/LTD data for the image recognition. The detailed fitting process of the parameters is described in Supplementary Note 1.

Material	Working Mechanism	Wafer Scale	Model of Operation	Nonlinearity of LTP/LTD	Ref.
$CsPbBr_3$ quantum dots	Charge trapping/detrapping	No	LTP: 365 nm<br>LTD: +10 V	LTP: 4.64<br>LTD: 2.73	2
In_2O_3/ZnO	Charge trapping/detrapping	No	LTP: 365±10 nm<br>LTD: -1 V	LTP: 3.49<br>LTD: 2.20	3
carbon dots/silk protein	Charge trapping/detrapping	No	LTP: 365 nm<br>LTD: -10 V	LTP: 1.64<br>LTD: 3.12	4
monolayer n-MoS_2	Charge trapping/detrapping	No	LTP: 310 nm<br>LTD: -8 V	LTP: 0.84<br>LTD: 2.23	5
amorphous InGaZnO	Charge trapping/detrapping	No	LTP: 375 nm<br>LTD: +8 V	LTP: 1.70<br>LTD: 1.33	6
C_3N_4	Charge trapping/detrapping	No	-	-	7
MoO_x	Hydrogen doping	No	-	-	8
VO_2	Photo-induced phase transition	Yes	LTP: 375 nm<br>LTD: -1 V~-3 V	LTP: 0.26<br>LTD: 1.11	This work

Supplementary Table 2. Device performance of the UV optoelectronic synapses.

Supplementary References

1. Zeiler, M. D.; Fergus, R., Visualizing and Understanding Convolutional Networks. *European Conference on Computer Vision* **2014,** *8689,* 818-833.
2. Wang, Y.; Lv, Z.; Chen, J.; Wang, Z.; Zhou, Y.; Zhou, L.; Chen, X.; Han, S. T., Photonic Synapses Based on Inorganic Perovskite Quantum Dots for Neuromorphic Computing. *Adv. Mater.* **2018,** *30* (38), e1802883.
3. Kumar, M.; Abbas, S.; Kim, J., All-Oxide-Based Highly Transparent Photonic Synapse for Neuromorphic Computing. *ACS Appl. Mater. Interfaces.* **2018,** *10* (40), 34370-34376.
4. Lv, Z.; Chen, M.; Qian, F.; Roy, V. A. L.; Ye, W.; She, D.; Wang, Y.; Xu, Z. X.; Zhou, Y.; Han, S. T., Mimicking Neuroplasticity in a Hybrid Biopolymer Transistor by Dual Modes Modulation. *Adv. Func. Mater.* **2019,** *29* (31), 1902374.
5. He, H. K.; Yang, R.; Zhou, W.; Huang, H. M.; Xiong, J.; Gan, L.; Zhai, T. Y.; Guo, X., Photonic Potentiation and Electric Habituation in Ultrathin Memristive Synapses Based on Monolayer MoS_2. *Small* **2018,** *14* (15), e1800079.
6. Duan, N.; Li, Y.; Chiang, H. C.; Chen, J.; Pan, W. Q.; Zhou, Y.X.; Chien, Y.C.; He, Y. H.; Xue, K. H.; Liu, G.; Chang, T. C.; Miao, X. S., An Electro-Photo-Sensitive Synaptic Transistor for Edge Neuromorphic Visual Systems. *Nanoscale* **2019,** *11* (38), 17590-17599.
7. Park, H. L.; Kim, H.; Lim, D.; Zhou, H.; Kim, Y. H.; Lee, Y.; Park, S.; Lee, T. W., Retina-Inspired Carbon Nitride-Based Photonic Synapses for Selective Detection of UV Light. *Adv. Mater.* **2020,** *32* (11), e1906899.
8. Zhou, F.; Zhou, Z.; Chen, J.; Choy, T. H.; Wang, J.; Zhang, N.; Lin, Z.; Yu, S.; Kang, J.; Wong, H. P.; Chai, Y., Optoelectronic Resistive Random Access Memory for Neuromorphic Vision Sensors. *Nat. Nanotechnol.* **2019,** *14* (8), 776-782.
